# Factors associated with unmet supportive care needs of oncology patients at Dessie Referral Hospital, 2020

**DOI:** 10.3332/ecancer.2021.1300

**Published:** 2021-10-05

**Authors:** Husniya Yasin Amane, Asressie Molla Tessema, Kemal Ahmed seid, Anissa Mohammed Hassen, Hussien Endris Assen, Zinet Abegaz Asfaw, Salih Mohamed endrie, Foziya Mohammed Hussien

**Affiliations:** 1 Department of Public Health, School of Public Health, College of Medicine and Health Science, Wollo University, Dessie 1145, Ethiopia; 2 Department of Anesthesia and Critical Care, College of Medicine and Health Science, University of Gondar, Gondar 196, Ethiopia; 3 Department of Anesthesia, College of Medicine and Health Science, Wollo University, Dessie 1145, Ethiopia

**Keywords:** unmet need, supportive care, oncology, Dessie

## Abstract

**Background:**

Assessment of supportive care needs for cancer patients and identifying factors affecting these needs is important for the implementation of supportive care programmes, as the burden of cancer is increasing in Ethiopia.

**Objective:**

To determine the prevalence and associated factors of unmet supportive care needs of cancer patients at Dessie Referral Hospital, Dessie, South Wollo, North East Ethiopia, 2020.

**Methods:**

A cross-sectional study design was implemented among 405 cancer patients from February to 30 July 2020, at Dessie Referral Hospital. The data were collected using a validated supportive care needs survey questionnaire through face to face interview and data extraction tools. Both descriptive and inferential statistics were used and bi-variable and multivariable logistic regressions were used to describe the association between dependent and independent variables. Thus, a p-value of less than 0.05 was considered statistically significant.

**Result:**

From the total 405 participants, 275 (67.5%) were females with a mean age of (mean ± standard deviation) 48.6 ± 15.4 years. Unmet supportive care needs were higher among psychological needs (81.0%, 95% (confidence interval) CI = 77.0–84.9) and physical needs (74.6%, 95% CI = 70.1–79.0). Old age was associated with unmet physical and psychological needs domain than young age (adjusted odds ratio (AOR) = 1.03; 95% CI: 1.01–1.06), (AOR = 1.06; 95% CI: 1.03–1.09), respectively. High household income was significantly associated with health information needs (AOR = 2.22; 95% CI: 1.33–13.93), remission status (AOR = 0.37; 95% CI: 0.22–0.62) was associated with patient/supportive care needs, late stage cancer was also significantly associated with physical, psychological and health information needs of patients (AOR = 2.19; 95% CI: 1.18–4.06), (AOR = 2.3; 95% CI: 1.18–4.57) and (AOR = 2:95%; CI: 1.03–3.86), respectively. Besides, source of information had a statistically significant association with psychological, health information and patient care needs domain (AOR = 2.61; 95% CI: 1.15–5.93), (AOR = 3.1; 95% CI: 1.65–5.82) and (AOR = 2.2; 95% CI: 1.25–3.87), respectively.

**Conclusion and recommendation:**

This study shows that the prevalence of unmet supportive care needs in cancer patients is high in each domain. Age, income, cancer stage, cancer site, treatment option, time since diagnosis and sources of information were associated across one or more unmet supportive care needs domains. Therefore, the government and health professionals should work together to improve the unmet needs of cancer patients.

## Introduction

In the past few decades, most countries have experienced a health transition that resulted in a dramatic shift in the disease burden from communicable and nutrition-related diseases to non-communicable diseases [1]. Among the global deaths in 2018, 63% were attributed to non-communicable diseases, and the toll is expected to increase further with the ageing of the population, urbanisation and globalisation of risk factors [2].

The global burden of cancer statistics has estimated 7.7 billion new cancer cases and 9.9 billion deaths in 2020 [3]. From the whole cancer data, Europe accounts for 23.4% of the global cancer cases and 20.3% of the cancer deaths, but it has only 9.0% of the worldwide population. On the other hand, the Americas have 13.3% of the global population and account for 21.0% of incidence and 14.4% of mortality worldwide. In contrast to other world regions, the proportions of cancer deaths in Asia and Africa are 57.3% and 7.3%, respectively [2]. In Ethiopia, cancer is the second top non-communicable disease next to cardiovascular disorders and its burden is aggravated by lack of early detection and timely treatment [4, 5].

The supportive care need is a broad term covering psychological, health system, physical or daily living, patient care or support, and sexuality needs [6]. Due to the disease’s progressive nature, supportive care is more important for cancer patients compared with patients with other non-communicable diseases. Various studies have shown that early initiation of supportive care significantly improves the survival and quality of life of cancer patients [7].

‘Unmet needs’ roughly represents the deficiencies in every area of patients’ lives, that arises due to having to deal with a diagnosis of a cancer diagnosis, which lack the level of service or support an individual perceives is necessary to achieve optimal well-being [8]. These needs can develop at any stage in the disease course, from diagnosis to the completion of treatment or death. Assessing the unmet supportive care need of cancer patients has a lot of advantages for both the patient and the government. It helps to prioritise the service to allocate resources depending on the urgency of the need and identify patient subgroups with higher-level needs for prevention or at least reduce problems through appropriate early intervention [9, 10]. According to different research conducted at general cancer population, 27%–60.2% of patients had a low to a high level of unmet supportive care needs [11–13]. Similarly, a study done in the United Kingdom stated that one-quarter of the patients reported unmet supportive care needs [14]. According to a survey done in Africa, nearly 46% of the participants indicated supportive care unmet needs [15].

Unmet needs have been categorised into these major domains: physical, psychological, informational, patient care and sexual needs [16]. Different researchers have found that the largest unmet needs are related to the psychological needs domain [17–19], and in much lesser frequency is found in patient care and sexual domains [20, 21]. A systematic review in Asia and Africa showed that psychological, physical and healthcare service/information domains were the three most commonly reported domains of unmet needs for cancer patients [15, 18]. A study conducted at the university of Gondar revealed that the overall mean score level of unmet need was 3.49, with health system and information need being the highest unmet needs [22].

The unmet needs of cancer patients and the level of satisfaction with the overall care were found to influence health related quality of life. Therefore, addressing the unmet needs of cancer patients and ensuring a higher satisfaction rate are recommended to maintain adequate health-related quality of life [23]. Predictors of unmet supportive care needs include younger age, advanced disease, lengthy cancer experience and anticancer treatments. Patients suffer from various problems, such as physical, psychological, emotional and practical issues [24].

Currently, the Ethiopian government has made efforts to prevent, control and manage cancer by implementing the Ethiopian National Cancer Control Plan in 2015 for the period of 2016–2020. In addition, the Ministry of Health set up five specialised cancer treatment centres in Gondar, Hawassa, Jimma, Tigray and Southern Nation and Nationality. However, the supportive care of cancer patients has not been considered a health priority and little is known about the supportive care need of oncology patients [25, 26]. Besides there has been only one study conducted in Ethiopia [22]; therefore, this study aims to determine the prevalence and associated factors of unmet supportive care needs of oncology patients at Dessie referral Hospital (DRH) in the only oncology Centre in North East Ethiopia.

## Methods

### Study design and setting

An institution-based cross-sectional study design was implemented on all cancer patients seen at DRH, Dessie, South Wollo Zone, Ethiopia from February to April 2020. DRH is one of the referral hospitals in the Amhara region with about 9 million catchment populations who came from more than 200 km away and is used as a teaching hospital for health science students. According to the Centre’s cancer registry, the hospital oncology centre started chemotherapy treatment for cancer patients in December 2018 and treated approximately 160 cancer patients per month.

### Data collection procedure and participants

The study populations were all patients diagnosed with cancer on any form of treatment for their disease at Dessie Referral Hospital. Patients who were severely ill and unable to communicate during the data collection period were excluded. Psychological needs, health information needs, physical or daily living, patient care and sexuality needs were the dependent variables. In contrast, socio-demographic characteristics (age, sex, educational status, marital status, employment status, residences, income, health insurances), information related factors (sources of information and informational status) and clinical variables (type of cancer, type of treatment, stage of cancer, time since diagnosis, recurrence history of chronic illness, remission status) were independent variables.

Those cancer patients who came for any type of treatment related to cancer during the data collection period were interviewed using a structured questionnaire adapted from different literatures and supportive care needs survey (SCNS) validated tool (See Appendix 1). The questions include the socio-demographic characteristics, informational status, clinical characteristics and patient’s supportive health care needs. Informational status was assessed as a total score composed of 10 different questions, which are basic information received on their diagnosis, prognosis, treatment is taken, medication benefit and side effects, duration of medication, a sequence of treatment, medical and tests a patient undergo the value might range from 0 to 10 maximum response [27, 28]. Data regarding patient’s clinical related factors were extracted from a patient card using a chart extraction checklist.

The original SCNS-short form (SF) 34 assesses cancer-specific perceived needs across five domains: Physical and daily living needs, psychological needs, health system information-needs, patient care and support needs and sexuality-needs. The participants were asked to indicate the level of their needs for the last month based on a 5-point Likert scale, The SCNS-SF has been validated at Hawassa referral hospital consisting of 25 items, which had overall Cronbach’s alpha of 0.933, ranging from 0.755 to 0.994 for the five domains [29]. For each item, participants could choose either ‘not applicable’ or ‘satisfied’ under the heading ‘no need’, or ‘low’, ‘moderate’ or ‘high’ need under the heading ‘some need’.

The data were collected by two BSc nurses working at DRH other than the oncology unit. The quality of data was maintained by giving training to data collectors about the questionnaire’s content, collecting data, study design, the significance of the study and the ethics of the research. Continuous monitoring and supervision were conducted by the principal investigator every day for completeness of the data. The questionnaire was translated to Amharic and then translated back to English for consistency. Besides, before the actual data were collected, a pre-test was conducted on 5% of the sample size for clarity and applicability of the tool, and feedback about the questionnaire.

### Data analysis procedure

The data were entered and coded into EpiData 4.2 software then exported to SPSS V26 software for analysis. Descriptive statistics and numerical summary measures were presented using frequencies distribution tables and graphs to describe the study population about relevant variables. For continuous variables, we use mean and median if the distribution is normal and skewed, respectively. The outcome variable, supportive health care needs, was recoded into ‘no needs’ and ‘some needs’. If a patient is reported as having at least one low to high need in a domain considered ‘unmet needs’ in that specific domain, and if a patient reports no need in all items to a single domain, it is considered as ‘no needs’ [30].

Bivariable logistic regression analysis with the help of odds ratio (OR) along with their 95% confidence interval (CI) was used to assess the degree of association between dependent and independent variables and variables whose (*p* < 0.25) was a candidate for multivariable logistic regression. Four independent models for each of the SCNS 25 domains were used to determine the association between independent variables and each of the domains (psychological, physical or daily living, health system, supportive needs domain). The statistical significance level was declared at a *p*-value < 0.05.

### Ethical consideration

This study was approved by the research and ethical committee of Wollo University College of Medicine and Health Science. Informed written consent was also secured from every study participant before the start of the study after telling them about the study’s objective. To ensure the confidentiality of the study participant’s information, anonymous typing was applied. So, the name of the participant and any identification of participants were not written on the questionnaire. All interviews were taken in a place that keeps privacy, and respondents chose the interview time.

## Results

### Socio-demographic characteristics of the participants

Out of 423 cancer patients seen at DRH during the data collection period, a total of 405 cancer patients aged 18 years or older were enrolled in the study giving a response rate of 95.7%. Of which 275 (67.9%) were females, and 268 (66.2%) were married. The mean age was 48.6 years (standard deviation (SD): 15.4) with a minimum of 18 and a maximum of 92 years. Two hundred eleven (52.1%) were unable to read and write, and 218 (53.8%) were jobless. More than half (54.1%) were from an urban area and 233 (57.3%) had health insurances coverage for their medical expenses.

More than one third of diagnosed cancer cases were breast cancer cases accounting for 138 (34.1%), followed by cervical cancer cases 77 (19.0%). Chemotherapy was found to be the leading treatment given for cancer patients accounting for 59.3%. Three hundred five (75.30%) were late-stage cancer cases. Eighty-nine (22%) had a history of remission, and 316 (99%) had recurrences. Furthermore, eighty-nine (22%) participants had a history of co-existing disease (Table 1).

### Information about diagnosis and related factors

Of the total patients, 344 (84.9%) have got information about their diagnosis. Two hundred six (50.9%) were informed about their current status of a disease. More than half 217 (53.6%) and 247 (61%) were informed about their possible cause of the disease and the medical test diagnosis of their disease, respectively. Three hundred sixteen (78%) and 349 (86.2%) were informed about their medical test and medical result, respectively. Three hundred forty-nine (86.2%) patients claimed that they were informed about the medical treatment they have taken, and 287 (70.9%) of patients had information about sequences of treatment they have received. Three hundred four (75.1%) were informed about the expected benefit, duration and possible side effects of the treatment. More than half 249 (59.5%) of patients received information from health professionals, and the rest were from health professionals and self-reading. The median and interquartile range (IQR) of patient informational status was 7 ± 1.6.

### The magnitude of the supportive care needs of participants

**Figure 1.**
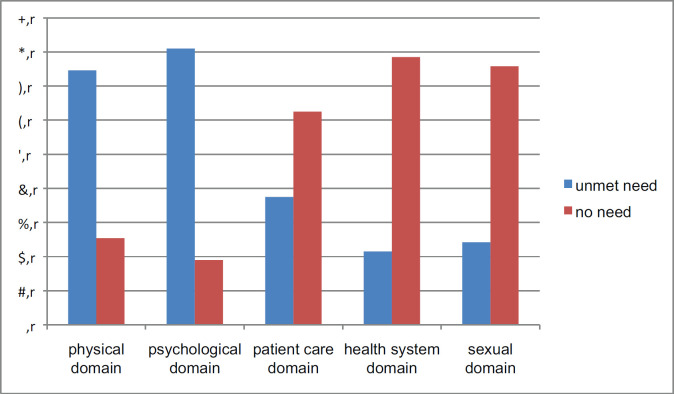
Magnitude of unsupportive care needs of an oncological patient, at Dessie Referral Hospital, Dessie, Ethiopia, 2020.

The most frequent need of patients was from the psychological domain accounting for 81% followed by physical or daily living needs 302 (74.6%). Finally, health system need was the least unmet supportive care need by patients accounting 87 (21.5%) (Figure 1).

### Factors associated with physical/daily living need domain

Binary and multivariable logistic regression analysis had been performed to assess the association between the dependent variable (physical needs domain) and independent variables by controlling the confounding variables.

In the binary logistic regression, age, occupational status, marital status, health insurances, household income, cancer site, cancer stage, treatment option, recurrences of diseases and coexisting diseases and informational status were significantly associated with the physical domain; hence further explored in the multivariable logistic analysis. As a result, age, cancer site, treatment option and stage of cancer become important predictors of physical need (*p* < 0.05) (Table 2).

For every 1-year increment in age, the odds of physical unmet needs increases by 3% (adjusted odds ratio (AOR) = 1.03; 95% CI: 1.01–1.06). The odds of physical needs were nine times more in patients with prostate and eight times more in lung cancer patients than breast cancer patients (AOR = 9.1; 95% CI: 2.34–35.34) and (AOR = 8.15; 95% CI: 1.76–37.6), respectively. Similarly, the odds of unmet physical needs were six times more in patients undergoing surgery compared with patients taking chemotherapy in the last 1 month (AOR = 6.92; 95% CI: 2.95–16.2). The odds of unmet physical needs were two times more in patients with end-stage cancer than patients who had early-stage cancer (AOR = 2.19; 95% CI: 1.18–4.06) (Table 2).

### Factors associated with psychological need domain

Binary logistic regression analysis revealed that, age sex, educational level, occupational status, presence of health insurance, household income, sources of information and informational status, cancer site, stage of cancer, treatment option, and time since diagnosis, remission and recurrences of disease were important candidates for the final model. However, only age, cancer site, stage of cancer and source of information become significant predictors in the multivariable logistic regression analysis (Table 3).

For every year increment in age, the odds of unmet psychological needs increased by 6% (AOR = 1.06; 95% CI: 1.03–1.09). The unmet psychological needs were seven times greater in patients diagnosed with prostate cancer when compared with patients diagnosed with breast cancer (AOR = 7.1; 95% CI: 1.3–39.6). Similarly, late-stage cancer patients had 2.3 times unmet psychological needs than early-stage cancer patients (AOR = 2.32; 95% CI: 1.18–4.57). Patients who had sources of information from both health professionals and self-reading had 2.6 times unmet psychological needs than those who had information only from health professionals (AOR = 2.61; 95% CI: 1.15–5.93). However, the unmet psychological needs were 0.09 times less among patients with a history of remission than no history of remission (AOR = 0.089; 95% CI: 0.036–0.22) (Table 3).

### Factors associated with health system information need

Variables significantly associated in the binary logistic regression were age, occupational status, monthly household income, cancer site, treatment option, stage of cancer, recurrences of diseases and time since diagnosis. However, in the multivariable logistic regression, only treatment option, stage of cancer, higher income and sources of information were significantly associated with health system information needs (Table 4).

The unmet health information needs were 68% less likely in patients who take analgesia than those taking chemotherapy (AOR = 0.32; 95% CI: 0.13–0.82). Similarly, unmet health information needs were two times more in patients who had end-stage cancer than patients who had early-stage cancer (AOR = 2; 95% CI: 1.03–3.86). In addition, patients who had household income of ≥2,700 ETB per month had 2.2 times unmet health system need. Moreover, patients who had sources of information from health professionals and self-reading had three times unmet health information needs than those who had information from only health professionals (AOR = 3.1; 95% CI: 1.65–5.82) (Table 4).

### Factors associated with patient care or supportive need

First cancer site, remission status, time since diagnosis and sources of information were significant predictors of patient or supportive care needs. The unmet patient care/supportive care needs were 63% less likely in patients who had a history of remission when compared with patients who had no history of remission (AOR = 0.37; 95% CI: 0.22–0.62). For every 1 month increase of time since diagnosis, the unmet patient care/supportive needs increased by 2% (AOR = 1.02; 95% CI: 1.00–1.04). Patients who had sources of information from health professionals and reading had more than two times unmet needs than patients who had sources of information only from health professionals (AOR = 2.2;95% CI: 1.25–3.87). Patients with skin cancer had three times unmet supportive needs than breast cancer patients (AOR = 2.53; 95% CI: 1.04–6.17) (Table 5).

## Discussion

The study assesses the prevalence and associated factors of unmet supportive care needs of oncology patients at Dessie Referral Hospital. The study revealed that unmet psychological and physical needs were the most unmet supportive care needs domains accounting 81.0% (95% CI = 77–84.9) and 74.6% (95% CI = 70.1–79.0), respectively. In addition, old age and household income, cancer site, treatment option, cancer stage, remission, time since diagnosis and sources of information were associated with one or more domains of unmet supportive care need.

The highest unmet supportive care need was recorded under the psychological and physical/daily living domain. Concerns about being able to feel about themselves, anxiety, feelings of sadness and fears about cancer spreading were paramount among unmet needs experienced by patients. The finding is comparable with other studies conducted in Nigeria, United Kingdom, Australia and United Arab Emirates [11, 19, 31, 32]. Emphasising sustained focus on Psycho-oncology treatment and the normal treatment for cancer patients will reduce the unmet needs of cancer patients.

The second top unmet need domain was related to the physical domain, accounting about 74.6%. This is similar to the Iranian study [33]. However, studies conducted in Indonesia and Denmark states as the first top unmet needs accounting (80.4%) and (40%), respectively [21, 34]. The explanation could be that cancer treatments, i.e. chemotherapy and surgery have serious side effects like tiredness, vomiting and unable to do normal activities. As a result, this makes them have high physical unmet needs.

The least unmet need was related to the sexuality domain. This is in line with studies conducted in Iran [35], Nigeria [32] and Malaysia [36]. However, a study conducted in the United States revealed that the sexuality domain was the most unmet needs of patients [17]. The discrepancy might be due to the cultural, religious and ethical differences of the two countries. Most of the patients in our country are culturally conservative and not ready to disclose information related to sexual behaviour. As a result, further qualitative study is needed to explore the actual sexual needs of the patients. Another reason may be patients may not consider sexual needs as important as other needs like psychological and physical needs.

Our study found that for every increment of age by 1 year, the odds of unmet psychological and physical needs increased by 6% and 3%, respectively. This is in line with studies conducted in Chicago and England stating older patients had the high unmet needs in physical/daily living and psychological need domain [37, 38]. On the contrary, other researches conducted in the UK and the USA claimed that young age was more prone to unmet supportive health care needs [11, 39]. This discrepancy could be explained by old patients are easily fatigued; they cannot resist the side effect of complex and long-term chemotherapy and surgery treatments. As a result, they may develop the unmet physical/psychological needs.

The study also showed high-income patients had high unmet health information needs. Contrary to this, a study done in Athens revealed that high income was less associated with unmet supportive care needs [40]. Economically stable patients may need better treatment options as they can afford the medical fee of private hospitals. However, as the treatment is available at public hospitals, high-income patients may be disappointed by the service delivery compared to private hospitals. Besides, patients who have sources of information from both physician and self-reading have unmet health information need. According to Ethiopia’s medical ethical book on Article 27 states, ‘On legitimate grounds, left to the discretion of the doctor, information about serious diagnoses and/or prognosis may be withheld unless the patient demands it’ [41]. As a result, physicians may hold necessary information related to a disease. So, a patient who had sources of information besides a physician will better understand the prognosis and unfavourable outcome of a disease, and they may develop unmet health system need. Another possibility could be the increasing number of oncology patients leading them to short consultation time with physicians to address all the patients’ questions resulting from unmet health information needs.

The current study revealed that prostate cancer patients were more likely to have unmet needs in the physical and psychological domains (AOR = 9.1; 95% CI: 2.34–35.7) and AOR = 7.17; 95% CI: 1.3–39.6) than breast cancer patients, respectively. Contrarily, a study done in Latin shows that breast cancer survivors reported greater unmet needs compared to both prostate and colorectal cancer survivors (OR 2.33–5.86) [42]. This may be related to most prostate cancer patients having tumour or pain around the gentile area. This makes them psychologically discomfort and unable to move freely to work their normal activities. As a result, patients may need support from another person.

Late-stage cancer patients were two times more likely to have an unmet physical, psychological and health information needs. Similarly in a study done in Malaysia, cancer survivors with an advanced-stage diagnosis had greater physical and psychological needs [43]. This may be related to patients with advanced disease who will have complicated and long-term treatment. The unexpected side effect and struggle for complex and length treatment lead them to have physical and psychological unmet needs. Besides, patients with late-stage cancer will have less survival probability. This finding implies early intervention, and rehabilitation treatment has great clinical importance for cancer patients.

The other variable significantly associated with psychological and patient care/supportive needs is the remission status. Patients with remission were less likely to develop unmet patient care needs (*p* < 0.001). This finding is similar with a Danish population-based study [34]. Patients with remission may adapt to the condition of the diseases and hospital environment. Besides, suppose a patient has repeated visits to the hospital. In that case, they may have access to get information and understand their disease condition, making a patient be psychologically ready and decrease their patient care/supportive needs.

Current study states that for every increase of time since diagnosis by 1 month, the odds of the unmet patient care and supportive needs increase by 2%. Similar findings have been reported in an Australian study [44]. The possible reason could be when the time since diagnosis is prolonged, the disease will progress late-stage increasing the severity of the illness. Thus, patients will develop to the verity of the disease increases leading them to develop patient care needs.

Generally, patients treated at DRH had high unmet supportive care needs, especially in psychological, physical and health information needs. Hence, different services and supports that address these needs should be prioritised to fulfil these needs by the patients.

## Strengths and limitations

This is the first study conducted at the only oncology centre of North-East Ethiopia to determine factors associated with unmet supportive care needs of oncology patients. As a result, its representativeness is high in the region. However, due to the study’s cross-sectional nature, the study could not show a cause and effect relationship. Besides, excluding patients who are unable to respond due to illness during the data collection may underestimate the specific need of those patients.

## Conclusion

This study revealed that the unmet supportive care needs of a patient within each domain are significantly high. Physical/daily living, psychological and health information/system were the topmost unmet need of cancer patients. In addition, age, income, education status, cancer site, treatment option, stage of cancer, remission and sources of information were associated with increased occurrences of unmet supportive care needs. Thus, the supportive care of cancer patients should be incorporated in the cancer treatment protocol. Furthermore, longitudinal and qualitative researches are better to measure the unmet need of cancer patients at different levels and exploring the unmet need of patients.

## Consent for publication

Not applicable.

## Availability of data and materials

All the necessary data are available in the main manuscript document and its supporting information file.

## Competing interests

The authors declare that they have no competing interests.

## Funding

Wollo University.

## Author’s contributions

AM, HY, AM and KA were involved in initiating the idea, write up of the proposal, data collection, data entry, data analysis and final manuscript write up. In contrast, FM, ZA, HE and SE were involved in the final manuscript editing and write up. Finally, all authors were involved in the approval of the final manuscript.

## Figures and Tables

**Table 1.** Socio-demographic and clinical characteristic of oncology patients at Dessie Referral Hospital, Dessie, Ethiopia, 2020.

Variables	Category	Number of participants *n* (%)
Mean age (±SD)	48.6 ± 15.4	
Sex of the patient	Male	130 (32.1)
	Female	275 (67.9)
Educational status	Unable to read and write	211 (52.1)
	Primary education	97 (24.0)
	Secondary education	46 (11.4)
	College and above	51 (12.6)
Occupational status	Employed	54 (13.3)
	Merchant	119 (29.4)
	Jobless/housewife	218 (53.8)
	Retired	14 (3.5)
Marital status	Single Married Divorced Widowed	41 (10.1) 268 (66.2) 34 (8.4) 62 (15.3)
Residence	Rural Urban	186 (45.9) 219 (54.1)
Health insurance	Yes	233 (57.5)
	No	172 (42.5)
Household income	≤1,500	155 (38.3)
	1,501–2,000	91 (22.5)
	2,001–2,700	60 (14.8)
	>2,700	99 (24.4)
First cancer site	Breast	138 (34.1)
	Colon	39 (9.6)
	Prostate	36 (8.9)
	Lung	37 (9.1)
	Skin	30 (7.4)
	Cervical	83 (19.0)
	Lipoma	42 (8.1)
Treatment options of the patients	Chemotherapy	240 (59.3)
	Surgery	84 (20.7)
	Analgesia	81 (20.0)
Stage of cancer	Stage I	14 (3.5)
	Stage II	86 (21.2)
	Stage III	162 (40.0)
	Stage IV	143 (35.3)
Remission of the disease	Yes	89 (22.0)
Recurrence of the disease	Yes	387 (99.5)
Co-existing disease	Yes	89 (22.0)
Median ± IQR**,** time since diagnosis (months)	4.0 ± 10	

SD, Standard deviation; IQR, Interquartile range

**Table 2.** Binary and multivariable logistic regression of variables associated with physical need domain among oncology patients at DRH, Dessie, Ethiopia, 2020.

Variables	Physical need domain				
	No need	Some need	COR (CI)	AOR (CI)	*p* value
**Age**			1.04 (1.01–1.05)	1.03 (1.01–1.06)	<0.001^a^
**Occupational status**					
Employed	15 (14.6)	39 (12.9)	1	1	
Paid work	32 (31.1)	87 (28.8)	1.04 (0.51–2.15)	1.33 (0.56–3.18)	0.903
Jobless	55 (53.4)	94 (54.0)	1.14 (0.58-2.22)	1.67 (0.66–4.3)	0.701
Retired	1 (1.0)	13 (4.3)	5.0 (0.6–41.63)	2.62 (0.22–30.9)	0.137
**Marital status**					
Single	12 (11.7)	29 (9.6)	1	1	
Married	76 (73.8)	192 (63.6)	1.04 (0.51–15)	0.75 (0.30–1.87)	0.904
Divorced	4 (3.9)	30 (9.9)	3.1 (0.89–10.7)	1.75 (0.41–7.51)	0.074
Widowed	11 (10.7)	51 (16.9)	1.92 (0.75–4.89)	0.92 (0.25–3.34)	0.173
**Health insurance**					
Yes	69 (67.0)	164 (54.3)	1	1	
No	34 (33.0)	138 (45.7)	0.58 (0.36–0.93)	0.75 (0.43–1.34)	0.095
**Monthly household income**					
0–1,500	50 (48.5)	105 (34.8)	1	1	
1,501–2,000	22 (21.4)	69 (22.8)	1.49 (0.83–2.68)	1.82 (0.85–3.9)	0.18
2,001–2,700	9 (8.7)	51 (16.9)	2.7 (1.23–5.91)	2.95 (1.19–7.3)	0.013^a^
>2,700	22 (21.4)	77 (25.5)	1.66 (0.93–2.98)	1.92 (0.77–4.8)	0.085
**Informational status**	-		1.18 (1.03–1.36)	1.17 (0.98–1.38)	0.074
**Cancer site**					
Breast	52 (50.5)	86 (28.5)	1	1	
Colon	10 (9.7)	29 (9.6)	1.75 (0.79–3.89)	1.23 (0.49–3.06)	0.167
Prostate	3 (2.9)	33 (10.9)	6.6 (1.94–22.02)	9.1 (2.34–35.74)	0.003^a^
Lung	2 (1.9)	35 (11.6)	10.58 (2.44–45.8)	8.15 (1.76–37.6)	0.002^a^
Skin	7 (6.8)	23 (7.6)	1.98 (0.79–4.95)	1.67 (0.6–4.63)	0.141
Cervical	18 (17.5)	65 (21.5)	2.18 (1.16–4.08)	2.23 (1.06–4.67)	0.014^a^
Lipoma	11 (10.7)	31 (10.3)	1.7 (0.79–3.67)	1.36 (0.54–3.44)	0.174
**Treatment type**					
Chemotherapy	75 (72.8)	165 (54.6)	1	1	
Surgery	9 (8.7)	75 (24.8)	3.7 (1.8–7.96)	6.92 (2.95–16.2)	<0.001^a^
Analgesia	19 (18.4)	62 (20.8)	1.48 (0.83–2.65)	1.28 (0.64–2.57)	0.184
**Cancer stage**					
Early stage (I, II)	38 (36.9)	62 (20.5)	1	1	
Late stage (III, IV)	65 (63.1)	240 (79.5)	2.2 (1.39–3.69)	2.19 (1.18–4.06)	<0.001^a^
**Recurrence**					
No	2 (1.9)	16 (5.3)	1	1	
Yes	101 (98.1)	286 (94.7)	0.35 (0.08–1.56)	0.57 (0.11–2.82)	0.171
**Coexisting diseases**					
No	88 (85.4)	228 (75.5)	1	1	
Yes	15 (14.6)	74 (24.5)	1.9(1.04–3.49)	1.41 (0.7–2.86)	0.088

COR, Crude odds ratio; AOR, Adjusted odds ratio; CI, Confidence interval

a Statistically significant at *p* < 0.05

**Table 3.** Binary and multivariable logistic regression of variables associated with psychological need domain among oncology patients at DRH, Dessie, Ethiopia, 2020.

Variables	Psychological need				
	No need	Some need	COR (CI)	AOR (CI)	*p* value
**Age**			1.04 (1.02–1.06)	1.02 (1.01–1.03–1.09)	<0.001
**Sex**					
Female	217 (66.2)	58 (75.3)	1	1	
Male	111 (33.8)	19 (24.7)	1.56 (0.88–2.75)	0.96 (0.46–1.98)	0.123
**Educational status**					
Unable to read and write	36 (46.8)	175 (53.4)	1	1	
Primary education	29 (37.7)	68 (20.7)	0.48 (0.28–0.84)	0.73 (0.34–1.56)	0.071
Secondary education	6 (7.8)	40 (12.2)	1.37 (0.54–3.47)	2.12 (0.59–7.53)	0.506
College and above	6 (7.8)	45 (13.7)	1.54 (0.61–3.89)	1.72 (0.39–7.51)	0.358
**Household monthly income**					
0–1,500	36 (46.8)	119 (36.3)	1	1	
1,501–2000	20 (26.0)	71 (21.6)	1.07 (0.58–1.99)	1.37 (0.63–2.97)	0.822
2,001–2,700	6 (7.8)	54 (16.5)	2.7 (1.08–6.84)	2.23 (0.75–6.63)	0.077
>2,700	15 (19.5)	84 (25.6)	1.69 (0.87–3.29)	1.4 (0.49–4.03)	0.120
**Health insurance**					
Yes	52 (67.5)	181 (55.2)	1	1	
No	25 (32.5)	147 (44.8)	0.59 (0.35–1.00)	1.08 (0.55–2.09)	0.592
**Source of information**					
Physician	50 (64.9)	191 (58.2)	1	1	
Physician and reading	13 (16.9)	85 (25.9)	1.71 (0.88–3.31)	2.61 (1.15–5.93)	0.011^a^
**Cancer site**					
Breast	39 (50.6)	99 (30.2)	1	1	
Colon	3 (3.9)	36 (11.0)	4.7 (1.37–16.25)	1.86 (0.48–7.23)	0.064
Prostate	2 (2.6)	34 (10.4)	6.6 (1.53–29.22)	7.17 (1.3–39.6)	0.011^a^
Lung	5 (6.5)	32 (9.8)	2.56 (0.91–6.94)	1.29 (0.39–4.22)	0.074
Skin	4 (5.2)	26 (7.9)	2.56 (0.84–7.81)	1.86 (0.52–6.63)	0.099
Cervical	14 (18.2)	69 (21.0)	1.94 (0.98–3.85)	1.05 (0.44-2.5)	0.057
Lipoma	10 (13.0)	32 (9.8)	1.26 (0.56–2.81)	0.76 (0.27–2.13)	0.571
**Treatment option**					
Chemotherapy	53 (68.8)	187 (57.0)	1	1	
Surgery	10 (13.0)	74 (22.6)	2.09 (1.01–4.34)	2.18 (0.92–5.21)	0.046
Analgesia	14 (18.2)	67 (20.4)	1.35 (0.71–2.6)	0.43 (0.18–4.57)	0.359
**Cancer stage**					
Early stage (I, II)	30 (39.0)	70 (21.3)	1	1	
Late stage (III, IV)	47 (61.0)	258 (78.7)	2.35 (1.38–3.99)	2.32 (1.18–4.57)	0.023^a^
**Recurrence**					
No	1 (1.3)	17 (5.2)	1	1	
Yes	76 (98.7)	311 (94.8)	0.24 (0.03–1.83)	0.3 (0.032–2.95)	
**Time since diagnosis**			0.98 (0.96–1.00)	0.98 (0.95–1.01)	0.067
**Remission**					
No	10 (13.0)	159 (48.5)	1	1	
Yes	67 (87.0)	169 (51.5)	0.16 (0.08–0.32)	0.089 (0.036–0.22)	<0.001^a^

COR, Crude odds ratio; AOR, Adjusted odds ratio; CI, Confidence interval

a Statistically significant at *p* < 0.05

**Table 4.** Binary and multivariable logistic regression of variables associated with health system information need domain among oncology patients at DRH, Dessie, Ethiopia, 2020.

Variables	Health system information need				
	No need	Some need	COR (CI)	AOR (CI)	*p* value
**Age**			1.01 (0.99–1.03)	1.01 (0.88–1.04)	0.876
**Occupational status**					
Employed	44 (13.8)	10 (11.5)	1	1	
Paid work	95 (30.2)	23 (26.4)	1.05 (0.46–2.4)	1.32 (0.52–3.33)	0.900
Jobless	169 (53.1)	49 (56.3)	1.27 (0.59–2.72)	1.66 (0.58–4.76)	0.528
Retired	9 (2.8)	5 (5.7)	2.44 (0.67–8.89)	2.62 (0.55–12.4)	0.175
**Household income**					
0–1,500	112 (35.2)	43 (49.4)	1	1	
1,501–2,000	78 (24.5)	13 (14.9)	1.8 (1.29–5.86)	0.59 (1.27–5.27)	0.002
2,001–2,700	53 (16.7)	7 (8)	2.0 (1.14–6.81)	1.5 (1.15–4.01)	0.003
>2,700	75 (23.6)	24 (27.6)	2.56 (2.46–9.49)	2.2 (1.33–13.93)	0.001
**Source of information**					
Physician	207 (65.1)	34 (39.1)	1	1	
Physician and reading	65 (20.4)	33 (37.9)	3.09 (1.78–5.38)	3.06 (1.63–5.76)	<0.001^a^
**Cancer site**					
Breast	106 (33.3)	32 (36.8)	1	1	
Colon	31 (9.7)	8 (9.2)	0.85 (0.35–2.04)	0.94 (0.36–2.41)	0.724
Prostate	25 (7.9)	11 (12.6)	1.45 (0.65–3.28)	1.49 (0.57–3.83)	0.363
Lung	29 (9.1)	8 (9.2)	0.91 (0.38–2.2)	0.9 (0.35–2.32)	0.840
Skin	23 (7.2)	7 (8.0)	1.00 (0.39–2.56)	0.94 (0.34–2.6)	0.986
Cervical	67 (21.1)	16 (18.4)	0.79 (0.4–1.55)	1.26 (0.58–2.74)	0.495
Lipoma	37 (11.6)	5 (5.7)	0.48 (0.16–1.23)	0.46 (0.15–1.37)	0.120
**Treatment option**					
Chemotherapy	187 (58.8)	53 (60.9)	1	1	
Surgery	57 (17.9)	27 (17.9)	1.67 (0.96–2.89)	1.88 (0.98–3.52)	0.067
Analgesia	74 (23.3)	7 (8.0)	0.33 (0.14–0.77)	0.3 (0.12–0.77)	0.01^a^
**Cancer stage**					
Early stage (I, II)	83 (26.1)	17 (19.5)	1	1	
Late stage (III, IV)	235 (73.9)	70 (80.5)	1.45 (0.81–2.61)	2 (1.03–3.86)	<0.001^a^
**Recurrence**					
No	10 (3.1)	8 (9.2)	1	1	
Yes	308 (96.9)	79 (90.8)	0.32 (0.12–0.84)	0.38 (0.13–1.12)	0.170

COR, Crude odds ratio; AOR, Adjusted odds ratio; CI, Confidence interval

a Statistically significant at *p* < 0.05

**Table 5.** Binary and multivariable logistic regression of variables associated with patient/supportive need domain among oncology patients at DRH, Dessie, Ethiopia, 2020.

Variables	Patient care/supportive need				
	No need	Some need	COR (CI)	AOR (CI)	p value
**Educational status**					
Unable to read and write	135 (53.4)	76 (50.0)	1	1	
Secondary not completed	61 (24.1)	36 (23.7)	1.05 (0.63–1.72)	1.23 (0.67–2.28)	0.853
Secondary education	29 (11.5)	17 (11.2)	1.04 (0.54–2.01)	0.74 (0.31–1.79)	0.905
College and above	28 (11.1)	23 (15.1)	1.46 (0.78–2.71)	0.87 (0.32–2.37)	0.232
**Marital status**					
Single	26 (10.3)	15 (9.9)	1	1	
Married	173 (68.4)	95 (62.5)	0.95 (0.48–1.88)	0.91 (0.40–2.08)	0.887
Divorced	16 (6.3)	18 (11.8)	0.95 (0.77–4.92)	1.5 (0.50–4.47)	0.157
Widowed	38 (15.0)	24 (15.8)	1.09 (0.48–2.47)	1.03 (0.35–3.0)	0.828
**Health insurance**					
Yes	157 (62.1)	76 (50.0)	1	1	
No	96 (37.9)	76 (50.0)	0.61 (0.41–0.92)	0.78 (0.8–1.25)	0.058
**Household income**					
0–1,500	96 (37.9)	59 (38.8)	1	1	
1,501–2,000	66 (26.1)	25 (16.4)	0.61 (0.35–1.08)	0.65 (0.35–1.21)	0.092
2,001–2,700	36 (14.2)	24 (15.8)	1.08 (0.59–1.99)	1.14 (0.56–2.33)	0.794
>2,700	55 (21.7)	44 (28.9)	1.3 (0.78–2.17)	1.31 (0.61–2.79)	0.313
**Sources of information**					
Physician	159 (62.8)	82 (53.9)	1	1	
Physician and reading	48 (19.0)	50 (32.9)	2.02 (1.05–3.25)	2.2 (1.25–3.87)	0.004^a^
Mixed	46 (18.2)	20 (13.2)	0.84 (0.47–1.52)	0.99 (0.51–1.91)	0.570
**Cancer site**					
Breast	96 (37.9)	42 (27.6)	1	1	
Colon	25 (9.9)	14 (9.2)	1.28 (0.6–2.7)	1.09 (0.48–2.5)	0.518
Prostate	19 (7.5)	17 (11.2)	2.04 (0.97–4.32	2.07 (0.88–4.87)	0.061
Lung	19 (7.5)	17 (11.2)	1.74 (0.82–3.67	1.72 (0.75–3.91)	0.144
Skin	21 (8.3)	16 (10.5)	2.0 (0.89–4.46)	2.53 (1.04–6.17)	0.011^a^
Cervical	50 (19.8)	33 (21.7)	1.51 (0.85–2.67)	1.61 (0.82–3.14)	0.157
Lipoma	26 (10.3)	16 (10.5)	1.41 (0.68–2.89)	1.62 (0.71–3.68)	0.353
**Treatment option**					
Chemotherapy	154 (60.9)	86 (56.6)	1	1	
Surgery	45 (17.8)	39 (25.7)	1.55 (0.94–2.57)	1.36 (0.76–2.43)	0.087
Analgesia	54 (21.3)	27 (17.8)	0.89 (0.52–1.52)	0.61 (0.32–1.17)	0.684
**Remission status**					
No	89 (35.2)	80 (52.6)	1	1	
Yes	164 (64.8)	72 (47.4)	0.49 (0.32–0.73)	0.37 (0.22–0.62)	0.001^a^
**Recurrence**					
No	6 (2.4)	12 (7.9)	1	1	
Yes	247 (97.6)	140 (92.1)	0.28 (0.1–0.77)	0.34 (0.11–1.03)	0.0174
**Time since diagnosis**			1.01 (0.99–1.03)	1.02 (1.00–1.04)	0.042^a^

COR. Crude odds ratio; AOR, Adjusted odds ratio; CI, Confidence interval

a Statistically significant at *p* < 0.05

## Appendix 1

### Information sheet

Name of the investigator: Husniya Yasin

Name of the organization: Wollo University

### Introduction:

This information sheet is prepared by the investigator from wollo university, department of public health whose main aim is to assess the prevalence of unmet needs of oncologic patients and factors associated with the unmet health care needs of oncology patients.

### Purpose of the research

the purpose of this research is to determine factors associated with unmet health care need of oncology patient.

### Voluntary participation

Your participation in this research is entirely voluntary. It is your choice whether to participate or not. Whether you choose to participate or not, all the services you receive as any member of this community will continue and nothing will change. If you choose not to participate in this research, you will be offered all the services that are routinely offered. You may change your mind later and stop participating even if you agreed earlier.

### Confidentiality

The information collected for this research will be kept confidential. Information about you that is collected during the research will be put away and no one but the researcher will be able to see it. Any information about you will have a number on it instead of your name. Only the researchers will know what your number is and Keep that information very secret that no one else can access, see or know it. It will not be shared with anyone.

### Benefits

this research may benefit you directly as an individual but it may have a benefit for health facilities for intervention.

### Risks and side effects

There are no side effects and known risks related to this of research so far.

#### Who to contact

This research will be reviewed and approved by the ethical review committee of Wollo University. If you wish to find about more or if you wish to ask questions now or later you can use the contact addresses below

Husniya Yasin 0913196525

### Consent Form

#### Greeting:

My name is ……………………………… I am here to collect information from you to know the health care need. Your participation in this research is entirely voluntary. It is your choice whether to participate or not. Whether you choose to participate or not, all the services you receive as any member of this community will continue and nothing will change. Information about you that is collected during the research will be put away and no one but the researcher will be able to see it. Your participation in this research may not directly provide you a certain benefit as an individual. It may benefit all mothers and children. There are no side effects and Known risks related to this kind of research so far and it takes only 10 minutes of participation. Up to now, you have been given all information that I feel you should know regarding the research project that you are being asked to participate in. I think you have understood the issues in detail. As I told you the survey has no risk, confidential, and takes only 10 minutes of interview.

Thank you for your cooperation and listening!!!

Are you willing to participate?

Yes No (stop the interview)

Name of data collector __________________ signature ____________

Name of Supervisor __________________ signature ____________

### Questionnaire

Code number _____

Date______________

### Part I Socio-demographic characteristics

**Table:**

Q.NO	QUESTIONNARIES	RESPONSE
01	Age	____________
02	Sex	male Femalle
03	Marital status	single married Divorced/separated Widowed Never married/single
04	Educational Level	anable to write and read Secondary not completed Secondary completed Trade/certificate/diploma Tertiary completed
05	occupation	employment Paid work Not working Retired
06	Income	______________
07	Resident	Urban Rural

### Part II Clinical Characteristics

**Table:**

07	Primary cancer site	Breast Colon and rectum Prostate Lung Skin/melanoma Don’t know Other
08	Treatment received in last month	Chemotherapy Radiotherapy Surgical removal of cancer Immunotherapy Hormone treatment Bone marrow treatment Othe
09	Time since diagnosis	------------------
010	Remission	yes no
011	Another illness other than cancer	yes no
012	Type of diseases	
012	Stage of cancer at time of diagnosis	I II III IV Unknown
013	Fear of recurrences	yes no
014	Treatment side effect	yes no

### Part III Information status about patient diagnosis

**Table:**

No	Questions	Coding categories	Skip
During your current disease or treatment, have you received information on:			
01	The diagnosis of your disease?	yes no	
02	The extent (spread) of your disease?	yes no	
03	The possible causes of your disease?	yes no	
04	The purpose of any medical tests you have had or may undergo	yes no	
05	The procedures of the medical tests?	yes no	
06	The medical treatment (chemotherapy, radiotherapy, surgery or other treatment modality)?	yes no	
07	The sequence of the medical treatments?	yes no	
08	The expected benefit of the treatment?	yes no	
09	The possible side-effects of your treatment?	yes no	
010	Where did you inform about the duration of your treatments?	yes no	
011	From whom did you hear about your diagnosis? Record all mentioned	health professional from a family member other patients other	

### Part IV supportive care need tool

**Instructions: This section of the questioner will assess supportive care needs, please circle the numbers or record on the space provided under the coding categories**

**Table:**

Physical need domain (5 items)		Coding category				
		No need		Unmet need		
No		**Not applicable**	**satisfied**	**Low need**	**Moderat need**	**High need**
1	Pain	1	2	3	4	5
2	Lack of energy/tiredness	1	2	3	4	5
3	Feeling unwell most of the time	1	2	3	4	5
4	Work around the home	1	2	3	4	5
5	Not being able to do the thing you used to do	1	2	3	4	5

Psychological domain		Coding category				
		No need		Unmet need		
		Not applicable	satisfied	Low need	Moderat need	High need
1	Anxiety	1	2	3	4	5
2	Feeling down or depression	1	2	3	4	5
3	Feeling of sadness	1	2	3	4	5
4	Fear about the cancer spreading	1	2	3	4	5
5	Worry that the results of treatment are beyond your control	1	2	3	4	5
6	Uncertainty about the future	1	2	3	4	5
7	Learning to feel in control of your situation	1	2	3	4	5
8	Keeping a positive outlook	1	2	3	4	5
9	Feeling about death and dying	1	2		4	5
10	Concern about the worries of those to you	1	2	3	4	5

Patient care/supportive need (3 items)		No need		Some need		
		Not applicable	satisfied	Low need	Moderate need	High need
1	Reassurance by medical staff the way you feel is normal	1	2	3	4	5
2	Hospital staff attending promptly to your physical need	1	2	3	4	5
3	Hospital staff acknowledging and showing sensitivity to your feeling and emotional needs	1	2	3	4	5

Health system/information (4 items)		No need		Some need		
		Not applicable	satisfied	Low need	Moderat need	High need
1	To be given explanations of those tests for which you would like explanations	1	2	3	4	5
2	To be adequately informed about the benefits and side effects of treatments before you choose to have them	1	2	3	4	5
3	To be informed about your test results as soon as possible	1	2	3	4	5
4	To be informed about cancer that is under control or diminishing (that is, remission)	1	2	3	4	5

Sexual domain (3 item)		Coding category				
		No need		Some need		
		Not applicable	Satisfied	Low need	Moderat need	High need
1	Change in sexual relationships					
2	Change in sexual feeling					
3	To be given information about Sexual relationships	1	2	3	4	5

## Amharic Version Questionnaire

የመረጃ ገፅ

የጥናቱ ዋና ተመራማሪ: ሁስኒያ ያሲን

የተቆሙ ስም - ወሎ ዩኒቨርስቲ

### መግቢያ

ይህ የመረጃ መስጫ ገፅ የተዘጋጀው የወሎ ዩኒቭረስቲ የህብረተሰብ ሳይንስ ድፓርትመንት ድፓርትመንት የጥናቱ ተመራማሪ በሆኑት ሲሆን የጥናቱም ዋና አላማ ያልተሞላ የጤና ፍላጎት አግልግሎት የካንሰር ታማሚወች መጠንና ተያያዥ ጉዳዪች በሚል ይሆናል፡፡

### የጥናቱም ዋና አላማ

የዚህ ጥናት ዋና አላማ ያልተሞላ የጤና ፍላጎት አግልግሎት የካንሰር ታማሚወች መጠንና ተያያዥ ጉዳዪች በሚል ይሆናል፡፡

### በፍቃደኝነት ላይ ተመሰረተ ተሳትፎ

የእርስወ በዚህ ጥናት ውስጥ መሳተፍ በፍቃደነትዎ ላይ ተመሰረተ ነው፡፡. በጥናቱ ለመሳተፍ ፈቃደኛ ባይሆኑ እንኮ ሲደረግልወት የነበረው ማንኛውም ነገር የሚቀጥል ይሆናል፡፡

### መተማመኛ

ከእርስዎ የሚገኘው ማንኛውም መረጃ በሚስጥር የሚጠበቅ ሲሆን በጥናቱ ቢድን አባል ብቻ የሚታይ ይሆናል፡፡. ለዚህም ሲባል የእርስዎ ሥምናአድራሻ አይገለጽም፡፡

### በጥናቱ በመሳተፍ የሚገኝ ጥቅም

እርስዎ በጥናቱ በመሳተፍዎ በግለሰብደረጃ የሚገኝ ጥቅም የሌለለ ሲሆንበሚደረገው ጥናት ግን ተቆም ተጠቃሚ ይሆናል፡፡

### በጥናቱ በመሳተፍ የሚመጣ ጉዳት

በጥናቱ በመሳተፍዎ ምንም አይነት ጉዳት አይደርስብወትም፡፡

ጥናቱን በተመለከተ ማንኛዉም አይነት ጥያቄ ካላችሁ የሚከተለዉን አድራሻ ተጠቀሙ::

በዋናነት ምርምሩን የሚያካሂዉ ሰዉ

ስም፡ሁስኒያ ያሲን

የስልክ ቁጥር- 0913196525

የስምምነት ውል

ሰላምታ

እኔ ……………………………… ይህ የመረጃ መስጫ ገፅ የተዘጋጀው የወሎ ዩኒቭረስቲ የህብረተሰብ ሳይንስ ድፓርትመንት ድፓርትመንት የጥናቱ ተመራማሪ አባል ሲሆን ሲሆን የጥናቱም ዋና አላማ ያልተሞላ የጤና ፍላጎት አግልግሎት የካንሰር ታማሚወች መጠንና ተያያዥ ጉዳዪች በሚል ይሆናል፡፡እርስወ በዚህ ጥናት ውስጥ መሳተፍ በፍቃደነትዎ ላይ ተመሰረተ ነው፡፡. በጥናቱ ለመሳተፍ ፈቃደኛ ባይሆኑ እንኮ ሲደረግልወት የነበረው ማንኛውም ነገር የሚቀጥል ይሆናል፡፡ ከእርስዎ የሚገኘው ማንኛውም መረጃ በሚስጥር የሚጠበቅ ሲሆን በጥናቱ ቢድን አባል ብቻ የሚታይ ይሆናል፡፡. ለዚህም ሲባል የእርስዎ ሥምና አድራሻ አይገለጽም፡፡ እርስዎ በጥናቱ በመሳተፍዎ በግለሰብደረጃ የሚገኝ ጥቅም የሌለለ ሲሆንበሚደረገው ጥናት ግን ተቆም ተጠቃሚ ይሆናል፡፡ ነገርግን እርስዎ በጥናቱ በመሳተፍዎ ምምን አይነት ጉዳት የማደርስብወት ይሆናል፡፡ለመጠየቅ 10 ደቂቃወች ብቻ የሚበቁን ሲሆን ጥናቱን በተመለከተ በቂ መረጃ ያገኙ ይመስለኛል፡፡

ስለተብብርዎ በቅደሚያ ከልብ አመሰግናለሁ፡፡

እርስዎ በጥናቱ በመሳተፍዎ ምምን አይነት ጉዳት የማደርስብወት ይሆናል፡፡

የዚህጥናት ዓላማው ገብቶኝ በጥናቱ ለመሣተፍ

ሀ. ፈቃደኛ ሆኛለሁ ለ. ፈቃደኛ አይደለሁም

በጥናቱ ለመሳተፍ ፈቃደኛ ከሆኑ ቃለ መጠይቁን መቀጠል ይቻላል፡፡

ፈቃደኛ ከሆኑ የመጠይቁ መለያ ቁጥር_____ መጠይቁ የተካሄደበት ቀን_______

የጠያቂው ሥምና ፊርማ________________

የሱፐርቫይዘር ስምና ፈርማ________________

### መጠይቆች

መለያ ቁጥር _____

ቀን______________

### ክፍል አንድ: ሶሽዎ ድሞግራፊክ ሁኔታ

**Table:**

ተ.ቁ	ጥያቄወች	መለስ
01	የተቀሙ ስም	
02	እድሜ	____________
03	ፆታ	1.ወንድ 2.ሴት
04	የጋብቻ ሁኔታ	1. ያላገባ 2. ያገባ 3. የተፋታ 4. ባለቤትዋ የሞተባት/የሞተበት 5. አግብቶ የማያቅ/አግብታ የማታውቅ
05	የትም/ት ደረጃ	1.ማንበብና መፃፍ የማይችል 2.ሁለተኛ ደረጃ ትም/ት ያላጠናቀቀ 3. .ሁለተኛ ደረጃ ትም/ት ያጠናቀቀ 4.ሰርቲፊኬት/ድፕሎማ 5.ድግሪ ወይም ከዚያ በላይ
06	የስራ ሁኔታ	1.ቅጥር 2.የግል ስራ 3.ስራ አጥ 4.በጡረታ የወጣ
07	የገቢ ሁኔታ	______________
08	የመኖሪያ ቦታ	1.ገጠር 2.ከተማ

### Part 2: የበሽታው ሁኔታ

**Table:**

08	የጤና መድህን አለወት	1.አዋ 2.
09	ካንሰሩ በቅድሚያ የጀመረበት የሰውነት ክፍል	1.ጡት 2.አንጀት 3.የዘር ፍሬ 4.ሳምባ 5.ቆዳ 6.አይታወቅም 7.ሌላ
010	በባለፈው አንድ ወር ያገኘው የህክምና ሁኔታ	1.ኬሞ ቴራፒ 2.ራዲወ ቴራፒ 3.የቀዶ ህክምና 4.ኢሚኒየዎ ቴራፒ 5.የሆርሞን ህክምና 6.የቦን ማሮው ህክምና 7.ሌሎች
011	በሽታው የታወቀበት ጊዜ	---------------
012	የመሻል ሁኔታ	1.አወ 2.አይደለም
013	ሌሎች ተያያዥ በሽታወች አሉ	1.አዋ 2.የለም
014	አው ካሉ ምን ምን	
015	በሽታው በታወቀበት ጊዜ የነበርው የካንሰሩ ደረጃ	1.I 2.II 3.III 04.IV 5.አያታወቅም
016	በሽታ ው መልሶ የመከሰት እድል	1.አለው 2.የለውም
017	ህክምናው የጎንዮሽ ጉዳት አለው	1.አዋ 2.የለዉም

### Part 3: የጥናቱ ተሳታፊዎች ስለ ህመማቸው ምን ያህል ተነግሮቸዋል፡፡

በህመምዎ ወይም በህክምናዎት ወቅት ምን መረጃ ከታች በተጠየቁት ጉዳዮች ተነግሮዎታል?

**Table:**

ተ.ቁ	ጥያቄዎች	መልሶችናየኮድመደቦች
01	ህመምዎምን እንደሆን ተነግሮዎታል?	1. አዎ 2. አልተነገረኝም
02	የህመምዎን ደረጃ እና ሁኔታ ተነግሮዎታል?	1. አዎ 2. አልተነገረኝም
03	የህመምዎ መንስኤ ምን ሊሆን እንደሚችልተነግሮዎታል?	1. አዎ 2. አልተነገረኝም
04	እስካሁን ያደረጉት የጤና ምርመራዎች ወይም ከዚህ በኃላየሚያደርጉትለምን እንደሆነ ተነግሮዎታል?	1. አዎ 2. አልተነገረኝም
05	የጤና ምርመራዎቹ እንዴት እንደ ሚካሄዱ ተነግዎታል?	1. አዎ 2. አልተነገረኝም
06	ለአርሰዎ ህመም ሊሆኑ የሚችሉ የህክምና አይነቶች እነማን እንደሆኑ ተነግሮዎታል? (ጨረር፤የቀዶ ጥገና ወይም ሌላ ህክምና አይነቶች)	1. አዎ 2. አልተነገረኝም
07	አንዱን ህክምና ሲጨርሱ የትኛውን ህክምና እንደሚቀጥሉስተነግሮዎታል?	1. አዎ 2. አልተነገረኝም
08	ህክምናዎቹ የሚሰጡት ጥቅሞች ተነግሮዎታል?	1. አዎ 2. አልተነገረኝም
09	በህክምናዎቹ ምክንያት ስሊኖሩ የሚችሉ የጎንዮሽ ጉዳቶችስ ተነግሮዎታል?	1. አዎ 2. አልተነገረኝም
010	ህክምናው ለምን ያክል ጊዜ ሊቆይ እንደሚችል ተነግሮዎታል?	1. አዎ 2. አልተነገረኝም
011	ስለ ህመመዎ እና አጠቃላይ ሁኔታ መረጃውችን ከየት ነው የሚያገኙት?	1. አዎ 2. አልተነገረኝም

### Part 4: የጥናቱ ተሳታፊዎች ምን ያክል የእገዛ እና እንእብካቤ በባለፈው ወር ውስጥ ያስፈልጋቸው ነበር?

**Table:**

ተ.ቁ	የአካልና የቀንከቀን ኑሮላይያለሁኔታ(5 ጥያቄዎች)	የኮድ መደቦች				
		ፍላጎት ያለ መኖር		ፍላጎት መኖር		
		ይሄችግር የለኝም	ፍላጎቴ ተሞልቶል	ትንሽ ፍላጎት አለኝ	መጠነኛ ፍላጎት አለኝ	ከፍተኛ ፍላጎትአ ለኝ
1	ህመም	1	2	3	4	5
2	የጉልበት ማጣት	1	2	3	4	5
3	የደህንነት ስሜት ማጣት	1	2	3	4	5
4	በቤት አካባቢ ስራ መስራት	1	2	3	4	5
5	መስራት የሚፍልጉትን አለመስራት	1	2	3	4	5
ሰነ-አይምሮ (10 ጥያቄዎች)		ፍላጎት ያለ መኖር		ፍላጎት መኖር		
		ይሄችግር የለኝም	ፍላጎቴ ተሞልቶል	ትንሽ ፍላጎት አለኝ	መጠነኛ ፍላጎት አለኝ	ከፍተኛ ፍላጎትአ ለኝ
1	ፍርሀት	1	2	3	4	5
2	የበታችነት ወይም የድብርት ስሜት	1	2	3	4	5
3	የመከፋት ስሜት	1	2	3	4	5
4	ወደሌላ ቦታ ይዛመትብኛል የሚል ፍርሃት	1	2	3	4	5
5	ህክምናወ ለውጥ አያመጣም ብሎ መጨነቅ	1	2	3	4	5
6	የተስፋ መቁረጥ ስሜት	1	2	3	4	5
7	በሥታው ከአቅሜ በላይ ነው ብሎ መጨነቅ	1	2	3	4	5
8	እድናለሁ ብሎ ማሰብ	1	2	3	4	5
9	እሞታለሁ ብሎ ማሰብ	1	2		4	5
10	ስለተፈጠረው ነገር አብዝቶ መጨነቅ	1	2	3	4	5
ህመምተኛውን ስለ መንከባከብና እገዛ (3ጥያቄዎች)		ፍላጎት ያለ መኖር		ፍላጎት መኖር		
		ይሄችግር የለኝም	ፍላጎቴ ተሞልቶል	ትንሽ ፍላጎት አለኝ	መጠነኛ ፍላጎት አለኝ	ከፍተኛ ፍላጎትአ ለኝ
1	በህክምና ቡድኑ መተማማን	1	2	3	4	5
2	የሆስፒታሉ ስታፎች ቅንነትና ትብብር	1	2	3	4	5
3	የሆስፒታሉ ስታፎች ቤተሰባዊ ድጋፍና ቅርበት	1	2	3	4	5
ከጤና ጋርየተገናኘ መረጃ (4 ጥያቄዎች)		ፍላጎት ያለ መኖር		ፍላጎት መኖር		
		ይሄችግር የለኝም	ፍላጎቴ ተሞልቶል	ትንሽ ፍላጎት አለኝ	መጠነኛ ፍላጎት አለኝ	ከፍተኛ ፍላጎትአ ለኝ
1	አንዲብራራሎት ስለሚፈልጉት የማርመራ አይነት ማብራሪያ አግንተዋል	1	2	3	4	5
2	የህክምናው አይነት ከመምረጥዋ በፊት ስለ ጥቅምና ተጓዳኝ ችግሮች ተነገርዎታል	1	2	3	4	5
3	ስለ ምርመራ ውጤትዋ መግለጫ ተሰጥተዋል	1	2	3	4	5
4	የታከሙበት ሀኪም ቤት የሚቻለውን ያክል አካዊ አርካታ የሚያኙበት ነበር	1	2	3	4	5
ስለሰነ-ጾታ (3 ጥያቄዎች)		ፍላጎት ያለ መኖር		ፍላጎት መኖር		
		ይሄችግር የለኝም	ፍላጎቴ ተሞልቶል	ትንሽ ፍላጎት አለኝ	መጠነኛ ፍላጎት አለኝ	ከፍተኛ ፍላጎትአ ለኝ
1	ከፍቅር አጋር ጋር ያለ ስሜት መቀነስ	1	2	3	4	5
2	የግብረስጋ ግንኙነት ፍላጎት መቀነስ	1	2	3	4	5
3	ስለ ፆታዊ ግንኙነት መረጃ አግኝተዋል	1	2	3	4	5

የተመራማሪው ስም--------------------------------------------

ፊርማ--------------------------

ቀን-------------------------

የጠያቂው ስም------------------------------------

ፊርማ----------------------------

ቀን-----------------------------
